# *In vitro* synergistic antifungal activity of silybin-posaconazole combination against *Candida auris*

**DOI:** 10.3389/fmicb.2026.1881511

**Published:** 2026-10-21

**Authors:** Mengqi Peng, Xiaolei Zhu, Qinwen Hu, Yuqing Fan, Yuqi Mao, Xinrou Chen, Hanbo Qin, Heng Zhang, Yi Sun

**Affiliations:** 1 Department of Dermatology, Hubei Provincial Clinical Research Center for Diagnosis and Therapeutics of Pathogenic Fungal Infection, Jingzhou Hospital Affiliated to Yangtze University, Jingzhou, Hubei, China; 2 Department of Clinical Medicine, Yangtze University, Jingzhou, Hubei, China; 3 Department of Ophthalmology, Jingzhou Hospital Affiliated to Yangtze University, Jingzhou, Hubei, China

**Keywords:** biofilm, *Candida auris*, posaconazole, silybin, synergistic effect

## Abstract

**Background:**

*Candida auris* infections pose a severe global public health threat. The hepatotoxicity of conventional azole antifungals and biofilm-mediated drug resistance adversely affect their clinical application. The natural component silybin (SLB) has both hepatoprotective and antimicrobial activities.

**Methods:**

This study was conducted in accordance with the CLSI M27-A4 standard, employing the broth microdilution plate method to evaluate the efficacy of SLB combined with itraconazole (ITR), voriconazole (VOR), posaconazole (POS) and fluconazole (FLC) against 10 *C. auris* strains. Synergistic effects were determined by measuring minimum inhibitory concentrations (MICs) and fractional inhibition concentration indices (FICIs). Potential synergistic mechanisms were investigated using ergosterol quantification, biofilm inhibition assay, reactive oxygen species (ROS) detection, Real-Time Quantitative PCR, SYTO9/PI double staining assay, and XTT assay.

**Results:**

Antifungal susceptibility testing showed that synergistic effects were most frequently observed when SLB was combined with POS (70% of strains), followed by SLB-VOR (50%) and SLB-ITR (30%). No synergism was detected for the SLB-FLC combination, while antagonism was observed in one strain for the SLB-ITR combination. The SLB-POS combination reduced ergosterol content, inhibited biofilm formation, and decreased the viability of preformed mature biofilms. It induced ROS accumulation, downregulated the expression of biofilm-associated genes (*bcr1*, *efg1*, *scf1*, *mkc1*, *erg2*, *erg11*, *als5*) under planktonic conditions, and suppressed biofilm proliferative activity.

**Conclusion:**

The SLB-POS combination exhibits synergistic antifungal activity against *C. auris.* Its activity is associated with multiple pathways, including elevated oxidative stress responses and reduced ergosterol content. In addition, this combination downregulates biofilm-related gene expression and impairs the capacity of planktonic cells to form biofilms. These findings provide preliminary experimental evidence for addressing *C. auris* drug resistance and support further investigation of this combination as a therapeutic strategy with an improved safety-efficacy profile and potential for clinical translation. Notably, the *in vitro* synergistic concentration exceeds the maximum oral bioavailable levels *in vivo*, and translational potential requires further *in vivo* verification.

## 1 Introduction

The global incidence of invasive fungal infections (IFIs) continues to increase, with *Candida* spp. among the leading fungal pathogens affecting immunocompromised patients. The incidence of *Candida*-related infections has increased significantly over the past three decades, primarily due to the AIDS epidemic, aging populations, growing numbers of immunocompromised patients, and the widespread use of medical devices (Horn et al., 2025; Silva et al., 2012). Among these pathogens, *C. auris*, an emerging multidrug-resistant (MDR) fungus, is inherently resistant to first-line azole antifungals such as fluconazole (FLC). The biofilm formed by *C. auris* can reduce fungal susceptibility to antifungals by 10- to 1,000-fold, leading to mortality rates of over 50% for invasive infections and posing a severe public health threat (Trofa et al., 2008). *C. auris* has a broad infection spectrum, ranging from superficial infections (e.g., vulvovaginal candidiasis, oropharyngeal candidiasis) to life-threatening systemic infections, such as invasive candidiasis (Czechowicz et al., 2022; McCarty et al., 2021). Biofilm formation is considered a major virulence factor of *Candida* spp., and is closely associated with a dramatic increase in resistance to most antimicrobial agents. *C. auris* biofilms exhibit enhanced resistance to antifungals, host defense responses, and physical stress compared to the planktonic fungal form (Cao et al., 2024; Hall and Mah, 2017).

In recent years, the development of new antimicrobial agents has slowed significantly, raising concerns about a potential “post-antibiotic era” in clinical practice (Salci et al., 2018). Traditional Chinese medicines (TCMs) have attracted increasing interest due to their abundant natural resources, relatively low cost, and favorable safety profiles. Numerous individual TCMs or compound preparations have demonstrated promising antifungal activity (YANG et al., 2025), suggesting the potential of TCM preparations in broadening antimicrobial spectra, reducing drug toxicity, limiting the emergence of drug-resistant strains, and addressing clinical drug resistance (Wang and Wu, 2018).

Silybin (SLB), a major bioactive flavonolignan isolated from the milk thistle *Silybum marianum*, is used both as a medicinal agent and a food supplement and has been widely applied in the treatment of various diseases, particularly liver disorders (Drouet et al., 2019; Gillessen and Schmidt, 2020; Post-White et al., 2007; Wang et al., 2020). As a flavonolignan, SLB possesses a typical lipophilic structure, characterized by multiple hydrophilic groups and hydrophobic aromatic rings (Figure 1). These structural features suggest a potential interaction with CYP51, the shared azole target, and raise the hypothesis that such interaction may contribute to SLB–POS synergism; this remains to be validated experimentally. SLB is documented to possess a range of beneficial properties, including anti-inflammatory, antioxidant, antifibrotic, antiviral, and cytoprotective effects, and its antioxidant activity is concentration-dependent (Angeli et al., 2010). Despite its well-established safety, SLB exhibits poor oral bioavailability (∼0.45%) and low aqueous solubility, which may limit its systemic exposure when administered orally (Li et al., 2025). As an antioxidant, SLB inhibits free-radical formation, scavenges reactive oxygen species (ROS), mitigates membrane lipid peroxidation, and increases the intracellular levels of antioxidant scavengers (Pereira-Filho et al., 2024). SLB has been shown to inhibit the growth of both Gram-positive and Gram-negative bacteria, as well as certain fungi, viruses, and protozoa, with the strongest antibacterial activity against *Staphylococcus epidermidis* (Xie, 2019).

**Figure 1:**
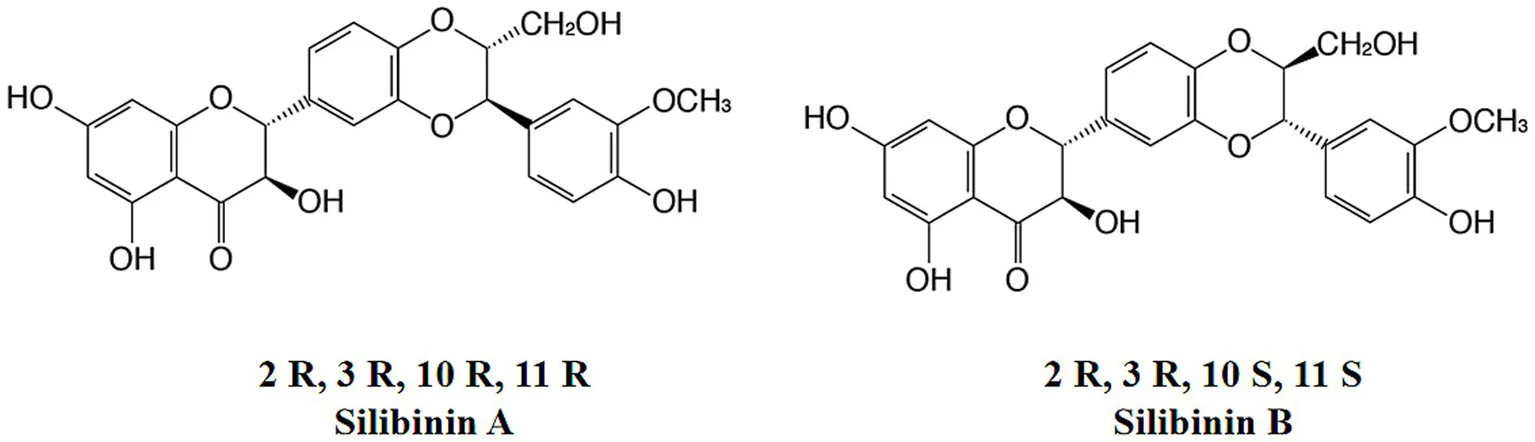
Chemical structure of the silibinin diastereoisomers, silibinin A and silibinin B (C_25_H_22_O_10_). Silybin is a flavonolignan. Its multiple hydroxyl groups, aromatic rings, and relatively planar backbone confer moderate lipophilicity and hydrogen-bonding capacity.

This study investigated the potential synergistic antifungal effects of SLB when combined with azole agents against *C. auris*. *In vitro* drug susceptibility testing, antibiofilm activity, cell viability, and potential molecular mechanisms were investigated at multiple levels. The study aimed to provide preliminary experimental evidence for the further development of combination therapies with improved efficacy and safety profiles against *C. auris.*

## 2 Materials and methods

### 2.1 Experimental strains

Ten *C. auris* strains were obtained from the Centers for Disease Control and Prevention (CDC), USA, and their identities were confirmed using morphological and molecular biological methods (Table 1) (Lockhart et al., 2017; Sharma and Kadosh, 2023; Vatanshenassan et al., 2020). Before each experiment, strains were activated on Sabouraud dextrose agar (SDA) at 37 °C for 2–3 days. *Candida parapsilosis* ATCC 22019 was used as the quality control strain.

**Table 1:** Information on clade, origin, and resistance mutations obtained from the CDC AR Bank, NCBI Pathogen Detection, and *Candida* Genome databases.

Strain	Source	*ERG11* mutation	Clusters according to other studies	Bio sample accession	Country
381	Ear		Cluster II (East Asia clade)	SAMN05379608	Japan
382	Wound		Cluster I (South Asia clade)	SAMN18754596	India
383	Blood	F126T	Cluster III (South Africa clade)	SAMN05379609	South Africa
384	Blood	F126T	Cluster III (South Africa clade)	SAMN05379610	South Africa
385	Blood	Y132F	Cluster IV (South America clade)	SAMN05379620	Venezuela
386	Blood	Y132F	Cluster IV (South America clade)	SAMN05379621	Venezuela
387	Blood	Y132F	Cluster I (South Asia clade)	SAMN05379624	Pakistan
388	Blood		Cluster I (South Asia clade)	SAMN05379577	Pakistan
389	Bronchoalveolar lavage (BAL) fluid		Cluster I (South Asia clade)	SAMN18754597	India
390	Pus		Cluster I (South Asia clade)	SAMN05379594	India

### 2.2 Reagents and instruments

SLB (batch No. 148-79-8, purity >99%), POS (batch No. 171228-49-2, purity >99%), voriconazole (VOR; batch No. 137234-62-9, purity >99%), itraconazole (ITR; batch No. 84625-61-6, purity >99%), and FLC (batch No. 129360, purity >98%) were purchased from Selleck Chemicals (USA). Stock solutions (1,600 μg/mL) were prepared in dimethyl sulfoxide (DMSO) and stored at −20 °C. The final DMSO concentration in all experiments was <0.1%. RPMI 1640 medium, 2,3-bis(2-methoxy-4-nitro-5-sulfophenyl)-2H-tetrazolium-5-carboxanilide (XTT), 2′,7′-dichlorodihydrofluorescein diacetate (DCFH-DA), and the SYTO9/propidium iodide (PI) live/dead staining kit were purchased from Sigma-Aldrich (USA). Hifair® III qPCR 1st Strand cDNA Synthesis SuperMix was obtained from Yeasen Biotechnology Co., Ltd. (China). The principal instruments used in this study included a Multiskan FC microplate reader (Thermo Fisher Scientific, USA), a FACSCanto II flow cytometer (BD Biosciences, USA), an LSM 880 confocal laser scanning microscope (CLSM) (Zeiss, Germany), and a LightCycler 480 Real-Time Quantitative PCR (RT-qPCR) system (Roche, Switzerland).

### 2.3 Azole antifungal susceptibility testing

Antifungal susceptibility was determined using the broth microdilution checkerboard method according to the CLSI M27-A4 standard and previously published protocols. The working concentration ranges of SLB, ITR, VOR, POS, and FLC were 1–128 μg/mL, 0.0625–8 μg/mL, 0.0625–8 μg/mL, 0.03125–4 μg/mL, and 0.0625–8 μg/mL, respectively (Chen et al., 2025).

The minimal inhibitory concentration (MIC) was defined as the lowest concentration that inhibited 50% of colony growth; this was calculated for each drug alone and in combination. The fractional inhibitory concentration index (FICI) was determined as: FICI = [MIC of Drug A (combination) / MIC of Drug A (alone)] + [MIC of Drug B (combination) / MIC of Drug B (alone)](When the MIC exceeded the highest tested concentration, twice the maximum tested concentration was used in the calculation; when it fell below the lowest tested concentration, half of the minimum tested concentration was used.). Interactions were classified as synergistic (FICI ≤ 0.5), no interaction (0.5 < FICI ≤ 4), or antagonistic (FICI > 4). All experiments were repeated three times on separate days (Tan et al., 2021).

### 2.4 Ergosterol quantification assay

Based on the *in vitro* antifungal susceptibility test results, we selected seven *C. auris* strains that exhibited synergistic effects with POS-SLB to investigate potential synergistic mechanisms. Yeast cell suspensions (1 × 10^8^ CFU/mL) were inoculated into 10 mL SAB broth containing drugs at their respective MICs, either alone or in combination, and incubated at 35 °C for 16 h with shaking. After centrifugation (3,000 rpm, 5 min), the pellet was washed twice with sterile distilled water, and its net wet weight was measured. Five hundred microliters of 25% ethanol-KOH solution (12.5 g KOH + 17.5 mL sterile water, made up to 50 mL with absolute ethanol) was added to the pellet, vortexed for 30 s, transferred to a sterile borosilicate tube, sealed, and incubated in an 85 °C water bath for 1 h. After cooling to room temperature, 150 μL of sterile water and 0.5 mL of n-heptane were added for sterol extraction, vortexed for 30 s, centrifuged (3,000 rpm, 5 min), and the heptane layer was transferred to a clean Eppendorf tube, after which the heptane was allowed to evaporate for 45 min to yield the sterol extract. The resulting sterol extract (20 μL) was mixed with 80 μL of absolute ethanol, and the absorption spectrum was recorded from 230 to 300 nm using a NanoDrop One spectrophotometer (Thermo Fisher Scientific). The ergosterol content was calculated as:


$$\begin{array}{l} \text{Ergosterol content}=\\ \;\;\{[(A281.5/290)\times F–(A230/518)\times F]/\mathrm{wet}\;\text{weight}\}\times 100\% \end{array}$$

where *F* is the dilution factor in absolute ethanol, and 290 and 518 are the E values (%/cm) of crystalline ergosterol and 24(28)-dehydroergosterol [24(28)DHE], respectively (Arthington-Skaggs et al., 1999).

### 2.5 Biofilm formation inhibition assay

The inhibitory effects of SLB and azole antifungal agents on biofilm formation were evaluated using crystal violet staining. The final concentrations of drugs used alone or in combination were determined according to the respective MIC values (see Table 2). A fresh yeast cell suspension was inoculated into SAB broth and incubated overnight at 30 °C with shaking (200 rpm) until reaching the logarithmic growth phase. Cells were harvested by centrifugation, washed three times with sterile phosphate-buffered saline (PBS), counted using a hemocytometer, and adjusted to 1 × 10^6^ CFU/mL in RPMI 1640 medium. One hundred microliters of the fungal suspension mixed with the drugs (serially diluted) were added to each well, and the plates were incubated at 37 °C with 5% CO₂ for 24 h to allow biofilm formation. The medium was then discarded and wells were washed three times with sterile PBS. Biofilm formation was evaluated using crystal violet staining, and absorbance was measured at 570 nm (OD₅₇₀) using a microplate reader. Inhibition rate was calculated as:


Inhibition rate(%)=[1–(OD1/OD0)]×100%

**Table 2:** *In vitro* interactions of silybin combined with azoles against 10 *Candida auris* isolates.

Combination	Isolates, *n*	Synergy, *n* (%) [exact 95% CI]	Indifference, *n* (%)	Antagonism, *n* (%) [exact 95% CI]	Synergy risk difference vs SLB-FLC, pp (exact conditional 95% CI)	Exact McNemar *P* for synergy vs SLBFLC	Holm-adjusted *p*
SLB-ITR	10	3 (30.0) [6.7–65.2]	6 (60.0) [26.2–87.8]	1 (10.0) [0.3–44.5]	30.0 (−12.5 to 30.0)	0.25	0.25
SLB-VOR	10	5 (50.0) [18.7–81.3]	5 (50.0) [18.7–81.3]	0 (0.0) [0.0–30.8]	50.0 (−2.2 to 50.0)	0.0625	0.125
SLB-POS	10	7 (70.0) [34.8–93.3]	3 (30.0) [6.7–65.2]	0 (0.0) [0.0–30.8]	70.0 (12.7 to 70.0)	0.015625	0.046875
SLB-FLC	10	0 (0.0) [0.0–30.8]	10 (100.0) [69.2–100.0]	0 (0.0) [0.0–30.8]	Reference	Reference	Reference

FICI, fractional inhibitory concentration index; pp, percentage points. Data are based on 10 *C. auris* isolates. Synergy was defined as FICI ≤ 0.50, indifference as 0.50 < FICI ≤ 4.00, and antagonism as FICI > 4.00. Exact 95% CIs for synergy, indifference, and antagonism were calculated separately using the Clopper–Pearson method; because these three outcomes are mutually exclusive, the intervals are marginal binomial CIs and may overlap. Because all combinations were tested against the same isolates, two-sided exact McNemar tests were used to compare the synergy rate of each SLB–azole combination with that of SLB–FLC, and Holm’s method was applied across the three comparisons. Synergy risk differences are absolute percentage-point differences versus SLB–FLC, and their exact conditional 95% CIs were derived from paired discordant results. Risk ratios and odds ratios were not estimable because SLB–FLC had no synergy events. For antagonism, SLB–ITR versus SLB–FLC had a risk difference of 10.0 pp (exact conditional 95% CI, −9.5 to 10.0; exact McNemar p = 1.000); no antagonism occurred with the other combinations.

where OD_1_ represents the experimental group, and OD_0_ represents the untreated control group (Wang et al., 2025).

### 2.6 ROS detection

Levels of ROS in planktonic fungi were measured using the DCFH-DA probe (Cao et al., 2024). Planktonic yeast cell suspensions (1 × 10^8^ CFU/mL) were mixed with SAB medium containing the DCFH-DA probe and drugs at their respective MICs, either alone or in combination. After incubation at 37 °C with shaking at 130 rpm, the cells were collected by centrifugation, washed with cold PBS, resuspended in PBS, and incubated according to the manufacturer’s instructions. Finally, cells were resuspended in cold PBS, and fluorescence intensity was measured within 30 min.

### 2.7 SYTO9/PI double-staining assay

The viability of biofilm-associated *C. auris* cells after drug treatment was assessed using the SYTO9/PI double-staining assay. The final concentrations of drugs used alone or in combination were determined according to the respective MIC values (see Table 2). After biofilm formation, planktonic cells were removed by three washes with cold sterile PBS. Drugs at their MICs, either alone or in combination, were added to each well, while blank control wells included 500 μL of blank RPMI 1640. Plates were incubated at 37 °C for 24 h, then washed three times with sterile PBS. A 1:1 mixture of SYTO9 and PI, prepared according to the manufacturer’s instructions, was added to each well, incubated at room temperature in the dark for 30 min, and observed under CLSM (Cao et al., 2024).

### 2.8 RT-qPCR assay

In this study, *tubA* was adopted as the internal reference gene. RT-qPCR was performed to analyze transcriptional alterations of biofilm-associated genes (*bcr1*, *efg1*, *mkc1*, *scf1*, *alns5*, *erg2*, *erg11*) after treatment with SLB combined with POS. RT-qPCR assays were performed on planktonic cells. After 24 h of drug treatment based on susceptibility results, the medium was aspirated using RNase-free pipette tips, and cells were collected by centrifugation. Blank control wells contained RPMI 1640 only. Cells were collected by centrifugation, washed once with DEPC-treated water, and the cell pellets were used for subsequent cDNA synthesis. cDNA was synthesized using HiFair®III qPCR 1st Strand cDNA Synthesis SuperMix. Three independent experiments were performed. Transcription levels were calculated using the comparative threshold cycle (ΔCT) method. Relative mRNA expression changes were determined by the 2^−ΔΔCT^ method (Livak and Schmittgen, 2001). All RT-qPCR primers are listed in Table 3 (Glazier, 2022; Harriott et al., 2010; Ponde et al., 2021; Ramage et al., 2002; Yang et al., 2025).

**Table 3:** Primer pairs and corresponding sequences.

Name	Seq (5′–3′)
auris-bcr1F	CCGCCACCGCCGTAACACAC
auris-bcr1R	CCGTCTGCTTGTGTGTGTCTGAG
auris-efg1F	CAGCACCAGCAGCAGTAC
auris-efg1R	GCAGCAGAAGAGTTGGAGGACCG
auris-scf1F	CCAAAGGGTGAACAGCCAGAAGG
auris-scf1R	ACAGAAGGAGCAGGAGCAGGTC
auris-mkc1F	ATGCTAACCTTGGTTCTCTGATTGACC
auris-mkc1R	AATGCTTCGTCCACGGTGATTCTC
auris-erg2F	AGGCTGACTTCTTGTTCAACGCCA
auris-erg2R	CCCAGTCAGCCTTCTTCTTAAT
auris-erg11F	GTGCCCATCGTCTACAACCT
auris-erg11R	TCTCTCTGCACAGCTCGAAA
auris-als5F	CAGGTTCGTCTTGTTCCCTATC
auris-als5R	TCTCTCGTTGTCGTTCATTCTG
auris-tubAF	TTGGTGATCTCGGCAACAGA
auris-tubAR	TCAGATGCCCAGTGACAGA

### 2.9 XTT assay

The effects of SLB and azole antifungal agents on the metabolic activity of *C. auris* during biofilm development were evaluated using the XTT reduction assay. The 90-min adhesion period was chosen to establish an early-stage biofilm prior to drug exposure, thereby evaluating the effects of the drugs on early biofilm development. Fungal suspensions were adjusted to 2 × 10^3^ CFU/mL in RPMI 1640 medium, and 100 μL of the suspension was added to each well of a 96-well plate. Following static incubation at 37 °C for 90 min to allow cell adhesion, the supernatant was removed, and the wells were washed three times with 100 μL of PBS. Then, 100 μL of RPMI 1640 medium containing the diluted drugs was added to each well, and the plates were incubated at 37 °C for 24 h with gentle shaking. After three further washes, 150 μL of XTT reagent was added to each well, followed by incubation for 3 h in the dark. Absorbance was measured at 490 nm (Koban et al., 2012).

### 2.10 Statistical analysis

Statistical analyses were performed using GraphPad Prism 10.1.20. Data are presented as mean ± SD from three independent biological replicates; technical replicates were averaged and were not treated as independent observations. Continuous data were analyzed using two-way ANOVA with strain and treatment as fixed factors and their interaction. When the interaction was significant, POS and POS + SLB were compared within each strain: Šidák correction was used for the ergosterol, biofilm, ROS, and XTT assays, whereas RT-qPCR was analyzed separately for each gene using Tukey’s test, with None used only for normalization. Exact adjusted *p*-values, effect sizes, and 95% CIs are reported; values below 0.0001 are reported as *p* < 0.0001. Antifungal combination rates were compared with SLB + FLC using paired exact McNemar tests with Holm adjustment, and proportions are reported with Clopper–Pearson 95% CIs. CLSM fluorescence was quantified using ImageJ. ns, *p* > 0.05; *p < 0.05; **p < 0.01; ***p < 0.001; ****p < 0.0001.

## 3 Results

### 3.1 Synergistic antifungal effects of SLB combined with azole agents

Although no antifungal efficacy was observed when SLB was used alone, SLB-azole exhibited significant synergistic effects. The highest synergy rate was observed for SLB-POS (7/10, 70.0%), followed by SLB-VOR (5/10, 50.0%) and SLB-ITR (3/10, 30.0%) (Table 2; Supplementary Table S1). Compared with SLB-FLC, only SLB-POS remained statistically significant after Holm adjustment. SLB-VOR and SLB-ITR showed numerically higher but not statistically significant synergy rates (see Table 2; Figure 2).

**Figure 2:**
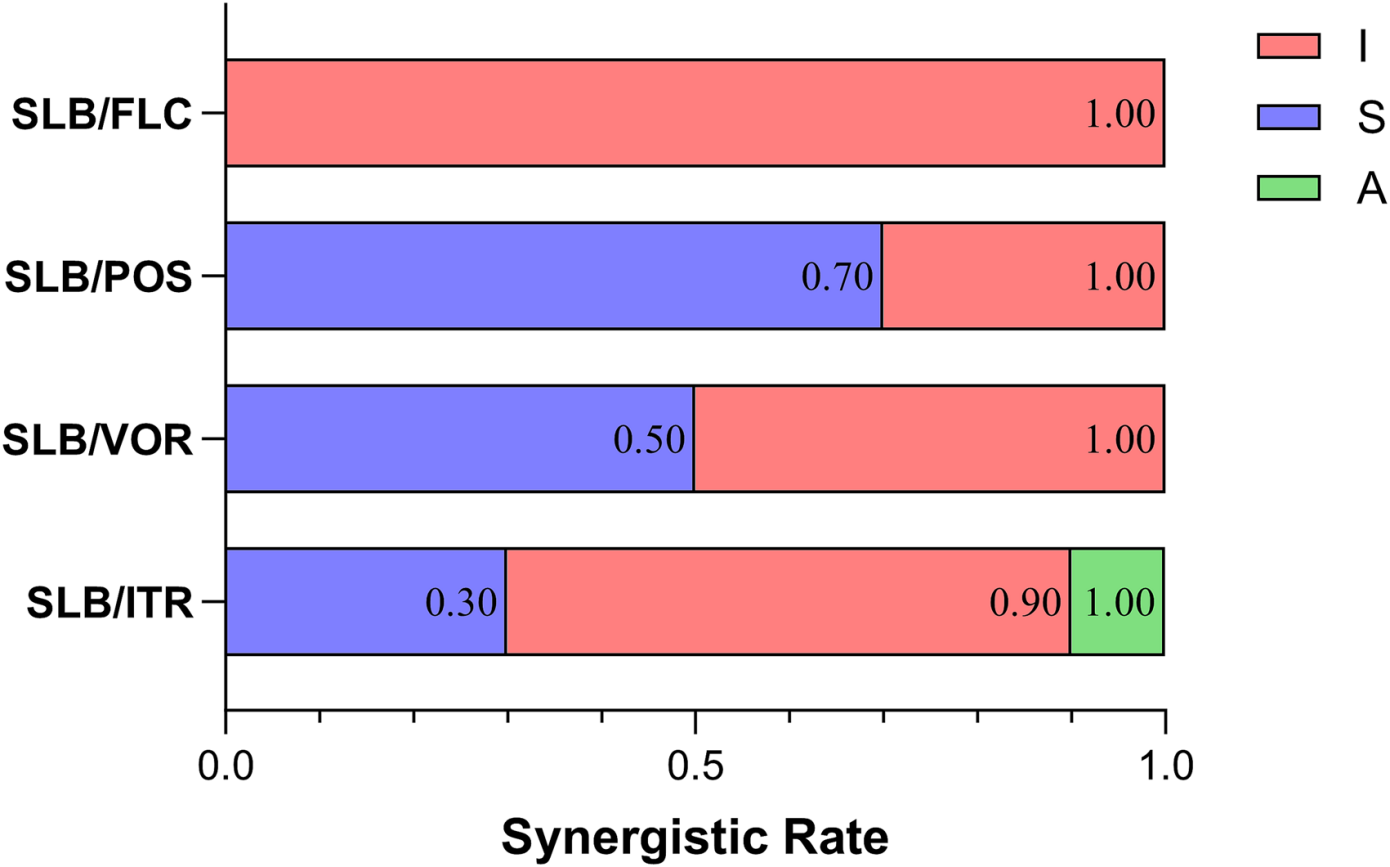
Combined susceptibility test results of SLB with ITR, VOR, POS, and FLC. SLB, silybin; ITR, itraconazole; VOR, voriconazole; POS, posaconazole; FLC, fluconazole. S, synergism (FICI ≤ 0.5); I, indifference (0.5 < FICI ≤ 4); A, antagonism (FICI > 4). MIC represents the minimum inhibitory concentration producing 50% growth inhibition. FICI, fractional inhibitory concentration index.

### 3.2 SLB combined with POS significantly reduces ergosterol content in *C. auris* strains

To explore the potential mechanism underlying the synergistic antifungal activity of the POS-SLB combination, ergosterol levels were quantified in eight *C. auris* strains. Strain 382, an indifference strain, showed no significant alteration in ergosterol content after POS - SLB treatment (*p* > 0.0609), whereas in the other seven strains, ergosterol levels were lower in the POS-SLB combination group than in the POS monotherapy group, with statistically significant differences (*p* = 0.0291 to *p* < 0.0001) (Figure 3, Supplementary Table S2).

**Figure 3:**
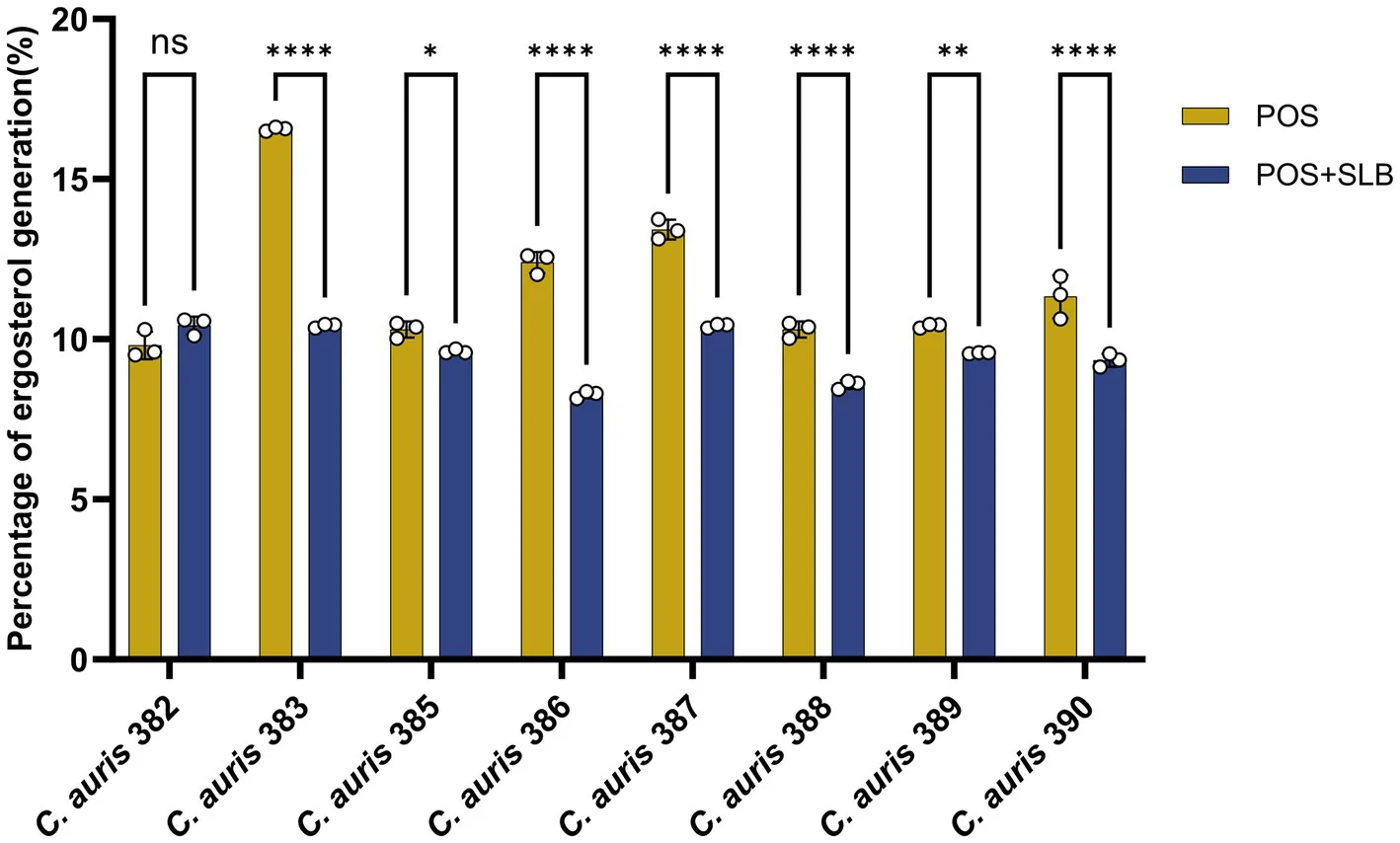
The SLB-POS combination reduces ergosterol content in *C. auris*. Ergosterol content was determined in seven *C. auris* strains after 16 h of treatment with POS alone or the SLB–POS combination under planktonic conditions. Ergosterol concentrations were quantified spectrophotometrically by measuring absorbance at 281.5 and 230 nm. Data are presented as the mean ± SD from three independent biological replicates, each with three technical replicates. Two-way ANOVA followed by Šidák’s multiple-comparison test was used. POS, posaconazole; SLB, silybin; ns, *p* > 0.05; *p < 0.05; **p < 0.01; ****p < 0.0001.

### 3.3 The SLB-POS combination inhibits biofilm formation in *C. auris*

Among the eight *C. auris* strains susceptible to synergistic antifungal activity, biofilm inhibition by POS alone ranged from 13.7 to 61.5%, with the least inhibition observed in *C. auris* 389 (13.7%) and the highest in *C. auris* 386 (61.5%). Combination treatment increased the inhibition rate to 24.2–79.7%. Except for strains 382 (*p* = 0.7859) and 390 (*p* > 0.2131), SLB combined with POS displayed greater biofilm-inhibitory activity than POS alone in the other six strains; the differences were statistically significant (*p* < 0.05), with inhibition improvements ranging from 16.5 to 46.5%. Notably, *C. auris* 385 exhibited the greatest enhancement in inhibition rate (Figure 4, Supplementary Table S3).

**Figure 4:**
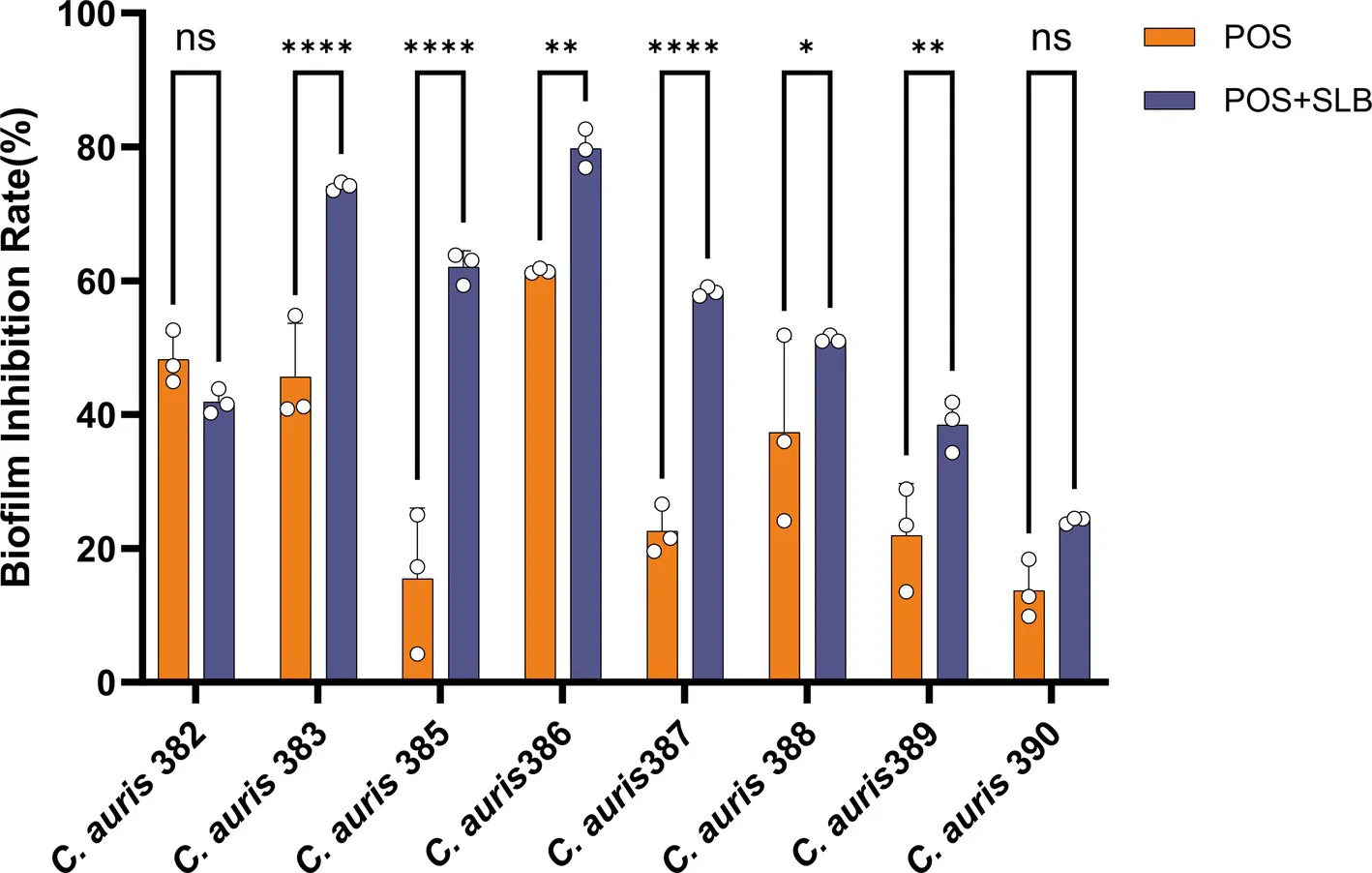
The SLB-POS combination enhances biofilm inhibition in *C. auris*. Biofilm formation was quantified using crystal violet staining after 24 h of incubation of 10 *C. auris* strains with POS alone or the SLB-POS combination at 37 °C. The treatment concentrations corresponded to the respective MIC values determined in the checkerboard assay. Rates of biofilm inhibition were calculated relative to the untreated control. Data are presented as the mean ± SD from three independent biological replicates, each with three technical replicates. Two-way ANOVA followed by Šidák’s multiple-comparison test was used. POS, posaconazole; SLB, silybin; ns, *p* > 0.05; *p < 0.05; **p < 0.01; ****p < 0.0001.

### 3.4 The SLB-POS combination is associated with increased ROS accumulation

Among the eight *C. auris* strains, except for strain 382 (*p* = 0.5865) and strain 387 (*p* = 0.0748), the ROS fluorescence intensity after SLB - POS combined treatment was higher than that after POS monotherapy in the remaining strains, and the differences were statistically significant (*p* = 0.0217). Notably, strain 386 showed the maximum fluorescence value (Figure 5, Supplementary Table S4).

**Figure 5:**
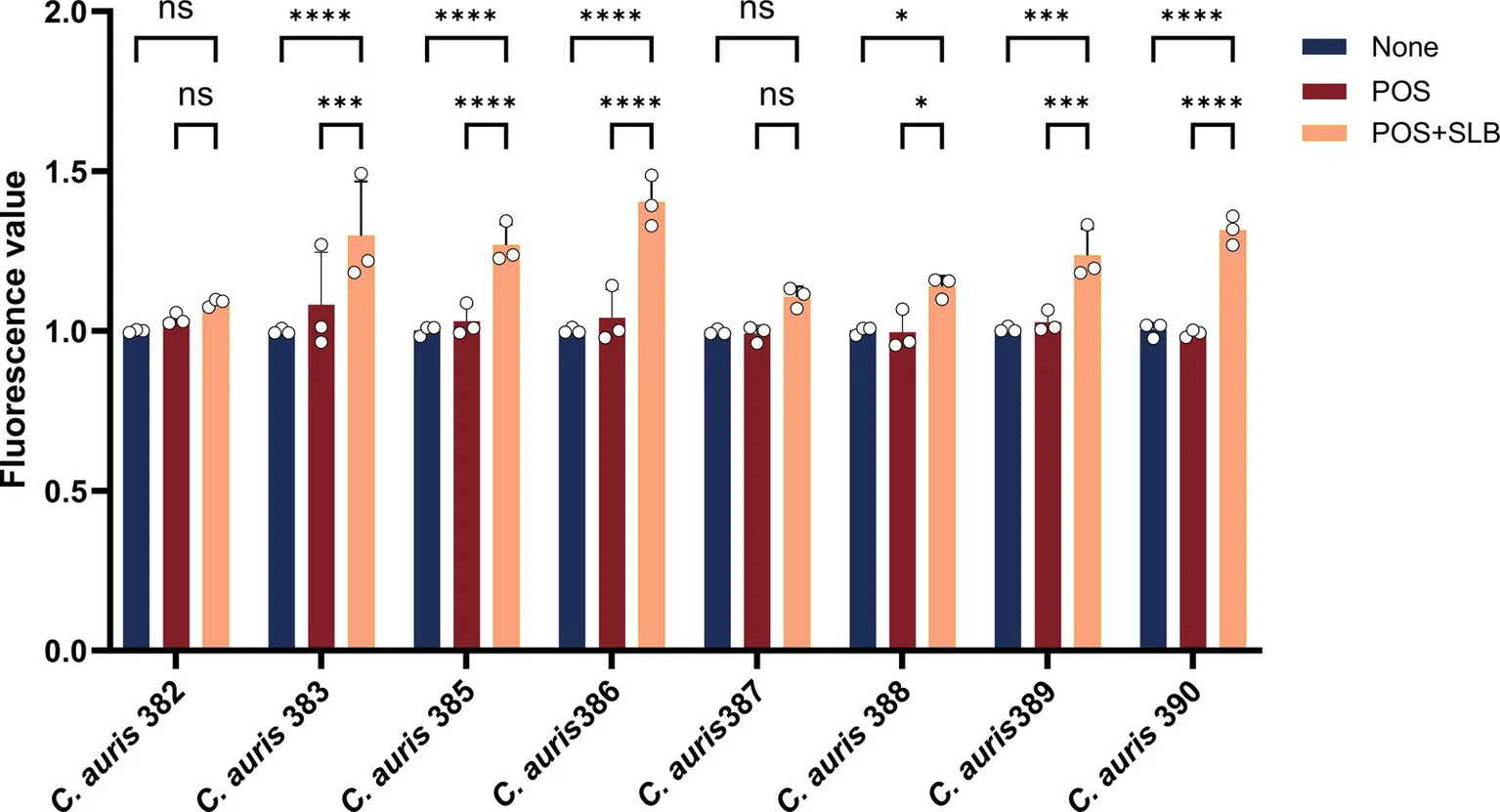
The SLB-POS combination is associated with increased ROS accumulation. Intracellular ROS levels were measured using the DCFH-DA probe in 10 *C. auris* strains after treatment with POS alone or the POS-SLB combination under planktonic conditions. ROS levels are presented as DCFH-DA fluorescence intensity compared with the untreated control group. Data are presented as the mean ± SD from three independent biological replicates, each with three technical replicates. Two-way ANOVA followed by Šidák’s multiple-comparison test was used. POS, posaconazole; SLB, silybin; ns, *p* > 0.05; *p < 0.05; ***p < 0.001; ****p < 0.0001.

### 3.5 Effects of the SLB-POS combination on biofilm-related gene expression in *C. auris*

RT-qPCR was performed to detect the expression levels of biofilm - related genes (*bcr1, efg1, mkc1, scf1, als5, erg2, and erg11*) in eight *C. auris* strains (seven synergistic strains and one non-synergistic strain) after treatment with POS alone or the POS - SLB combination. Among the seven synergistic strains, compared with the POS monotherapy group, SLB - POS combination treatment significantly down - regulated the expression of *bcr1*, *mkc1, erg2,* and *als5* in all seven synergistic strains. The *efg1* gene was down - regulated in six strains except *C. auris* 389 (*p* = 0.3846); the *erg11* gene was down - regulated in six strains except *C. auris* 390 (*p* = 0.1732); and the *scf1* gene was down-regulated in five strains except *C. auris* 389 (*p* = 0.4986) and *C. auris* 390 (*p* = 0.575). For strain 382, compared with the POS monotherapy group, the SLB - POS combination did not significantly alter the expression of the remaining tested genes, except for *mkc1* (*p* < 0.0001) and *als5* (*p* = 0.0008) (Figure 6, Supplementary Table S5).

**Figure 6:**
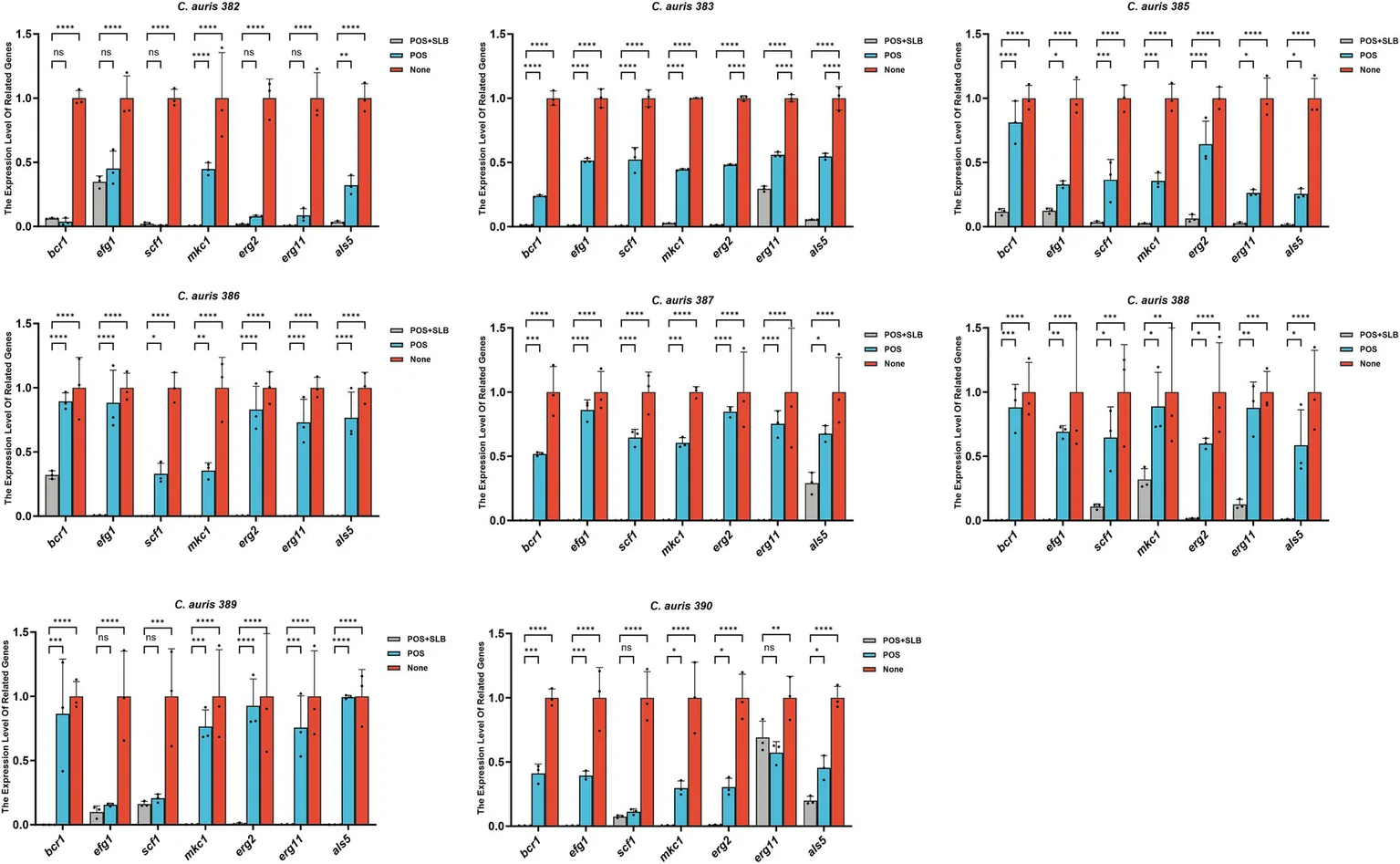
The SLB-POS combination regulates biofilm-related gene expression in *C. auris*. Relative expression levels of biofilm-related genes were determined by RT-qPCR in 10 *C. auris* strains after 24 h of treatment with POS alone or the POS-SLB combination under planktonic conditions. Gene expression was normalized to the reference gene *tubA* and quantified using the 2^−ΔΔCT^ method. Data are presented as the mean ± SD from three independent biological replicates, each with three technical replicates. Statistical analyses were performed using two-way ANOVA followed by Tukey’s *post hoc* test. ns, *p* > 0.05; *p < 0.05; **p < 0.01; ***p < 0.001; ****p < 0.0001.

### 3.6 Effects of the SLB-POS combination on the viability of *C. auris* biofilms, as shown by CLSM

The ratio of green to red fluorescence was used to assess biofilm cell viability following drug treatment. In the POS monotherapy group, the SYTO9/PI fluorescence ratio indicated limited antifungal activity against mature biofilms, with relatively low cell mortality. In contrast, treatment with the SLB - POS combination led to significantly decreased green fluorescence with increased red fluorescence, indicating reduced biofilm viability and increased cell death. These observations demonstrate that the SLB - POS combination is more effective than POS alone at killing *C. auris* biofilm cells, consistent with the synergistic antibiofilm activity observed in the quantitative assays (Figure 7).

**Figure 7:**
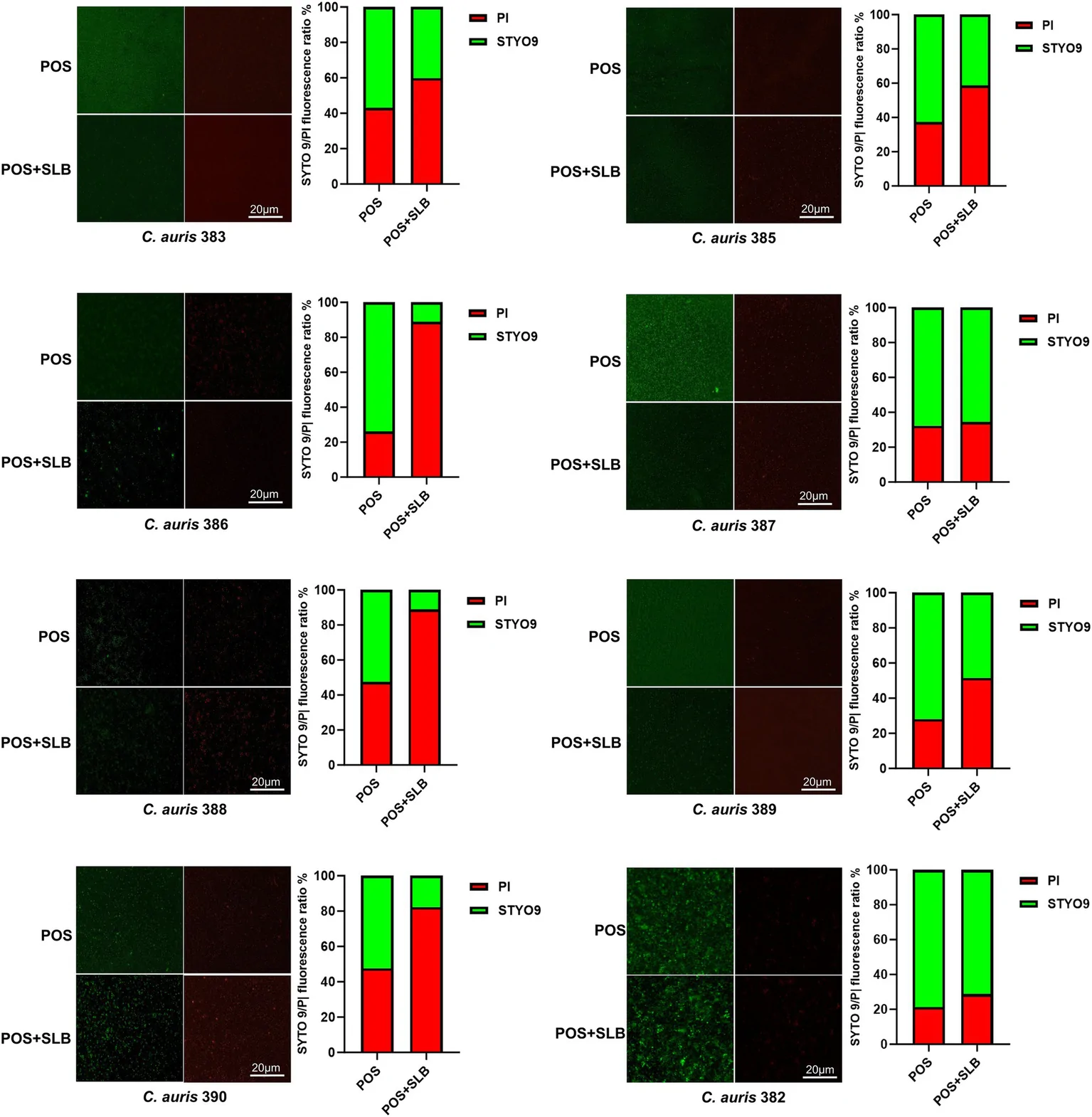
Effects of the SLB–POS combination on the viability of *C. auris* biofilms, as shown by CLSM. Biofilm viability was evaluated by SYTO9/PI double staining and CLSM. Mature biofilms were established for 24 h and then treated with POS alone or the SLB-POS combination for a further 24 h. SYTO9 (green) stains viable cells, whereas PI (red) stains nonviable cells. Scale bar = 20 μm. The bar graph shows the SYTO9/PI fluorescence ratio for each treatment group. Representative images from three independent experiments are shown. POS, posaconazole; SLB, silybin.

### 3.7 Biofilm metabolic activity of SLB-POS combination by XTT assay

The XTT reduction assay was used to evaluate the effects of POS alone and in combination with SLB on the metabolic activity of *C. auris* biofilms. As shown in Figure 8, both the POS monotherapy group and the combination group reduced the metabolic activity of *C. auris*. Among the eight strains, no significant difference in metabolic activity was observed between the monotherapy and combination groups for *C. auris* 382 (*p* = 0.9327), 389 (*p* = 0.9871), and 390 (*p* = 0.0524). For the remaining five strains, the combination group showed significantly greater inhibition of biofilm metabolic activity than the POS monotherapy group (*p* < 0.0001), indicating lower residual metabolic activity and enhanced inhibition after combination treatment (Figure 8, Supplementary Table S6).

**Figure 8:**
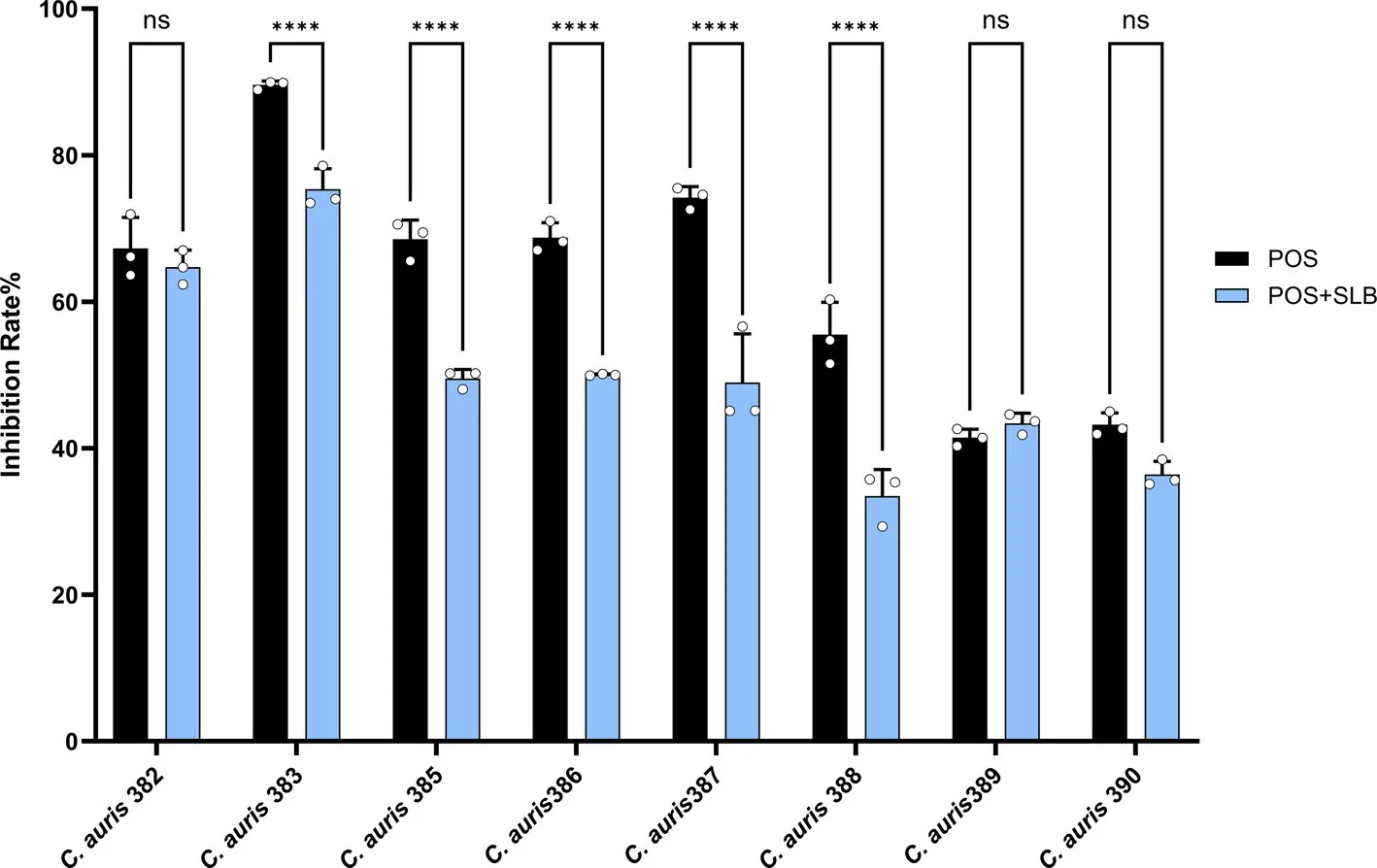
The SLB–POS combination reduces the metabolic activity of *C. auris* biofilms, as shown by the XTT reduction assay. Biofilm metabolic activity was assessed using the XTT reduction assay after treatment with POS alone or the SLB–POS combination for 24 h at 37 °C. Metabolic activity is expressed as a percentage of the untreated control. Data are presented as the mean ± SD from three independent biological replicates, each with three technical replicates. Two-way ANOVA followed by Šidák’s multiple-comparison test was used. POS, posaconazole; SLB, silybin; ns, *p* > 0.05; ****p < 0.0001.

## 4 Discussion

The core finding of this study is that the combination of the natural product SLB with the azole antifungal agent POS results in synergistic antifungal activity against *C. auris*. The SLB-POS combination reduced ergosterol content, was associated with altered membrane integrity and increased ROS accumulation in *C. auris*. Meanwhile, the combination inhibited biofilm formation, reduced the metabolic activity of biofilms and downregulated the expression of biofilm-related genes. These results suggest that the antifungal activity of this combination is associated with oxidative-stress-mediated fungal cell damage and antibiofilm effects. These findings suggest that the SLB–POS combination represents a promising strategy for addressing MDR *C. auris* infections while potentially reducing the hepatotoxicity associated with prolonged azole therapy.

*C. auris* represents a public health threat because of its ability to cause difficult-to-treat infections and hospital outbreaks, with mortality rates reaching up to 60% (Magobo et al., 2014). Due to its broad-spectrum resistance, conventional antifungal drugs display poor therapeutic effectiveness against *C. auris* infections. In this context, combination therapy has emerged as a promising alternative strategy to overcome drug resistance and improve treatment outcomes.

SLB, a major bioactive constituent of *Silybum marianum*, has long been used in the management of liver diseases. SLB has been shown to possess significant hepatoprotective potential, exerting anti-inflammatory and antioxidant effects by regulating the NF-κB signaling pathway and NLRP3 inflammasome activation (Ma K. L. et al., 2020). Both *in vitro* and animal studies have demonstrated that SLB effectively protects hepatocytes from toxin-induced damage and mitigates azole-induced hepatotoxicity (Al-Anati et al., 2009). The results of our checkerboard assay indicated that: although SLB alone exhibited no antifungal activity, the SLB-POS combination demonstrated stable synergistic inhibition against seven synergistic *C. auris* strains identified; in contrast, the synergistic effects of SLB combined with VOR or ITR were observed only in a subset of strains. This may be related to the molecular structure of POS, which has been reported to have stronger affinity for CYP51 (Chen et al., 2010). CYP51 is a key rate-limiting enzyme in the fungal ergosterol biosynthesis pathway and is thought to be closely associated with the fluidity, integrity, and stability of fungal cell membranes through ergosterol synthesis (Zhang et al., 2017). Our ergosterol quantification experiments indicate that SLB-POS combination treatment was associated with further reduction in cellular ergosterol levels compared with POS monotherapy. It is hypothesized that SLB and azoles may share a core target, namely, the sterol 14α-demethylase subtype of the fungal cytochrome P450 (CYP450) enzyme superfamily (Bédard et al., 2024; Sridar et al., 2004). However, whether SLB can enhance POS via CYP51 remains a hypothesis that requires further experimental validation.

Biofilm formation is a key virulence trait of *C. auris* and constitutes a major driver of acquired antifungal resistance (Kean and Ramage, 2019). Biofilm-mediated tolerance is up to 1,000 times higher than that of planktonic cells, thereby protecting *C. auris* from damage caused by triazole compounds (Fan et al., 2022). Biofilm-associated tolerance frequently causes persistent therapeutic failure. Based on the aforementioned drug sensitivity results, we employed three experimental models to comprehensively assess the anti-biofilm activity of SLB combined with POS. These models corresponded to different stages of biofilm development: inhibition of initial biofilm formation (crystal violet assay), inhibition of the metabolic activity of cells within the biofilm (XTT assay), and reduction of pre-formed mature biofilms (SYTO9/PI staining). The first two methods collectively confirm that the combination of SLB and POS exhibits stronger anti-biofilm activity compared to POS alone. This combined regimen not only inhibits the early formation of biofilms but also decreases the metabolic activity of cells in mature biofilms. This study also demonstrated the effectiveness of the synergistic antifungal combination in reducing the viability of pre-formed, mature biofilms, as visualized by CLSM following SYTO9/PI staining to assess the ratio of live to dead fungal cells (Ma S. et al., 2020). Compared with POS monotherapy, the combination of SLB and POS exerts more potent disruptive effects on *C. auris* biofilms and greater damage to fungal cell membranes, and markedly increases the proportion of dead fungal cells within biofilms. These findings are consistent with the quantitative crystal violet and XTT assay results. Three sets of experiments collectively verified from the perspectives of early biofilm formation, cellular metabolic activity within biofilms, and microscopic morphology that the anti-biofilm effect of the SLB-POS combination was markedly superior to POS monotherapy. This combination can not only inhibit biofilm formation, but also disrupt the structure of mature biofilms, damage fungal cell membranes, and eliminate fungal cells within biofilms.

To further investigate the molecular mechanisms underlying the anti-biofilm properties and synergistic effects of SLB and POS, we employed RT-qPCR to quantitatively analyze genes related to adhesion, biofilm formation, cell wall integrity, and ergosterol biosynthesis in *C. auris*. Genes were selected according to their previously documented roles. The adhesion of fungal cells is central to biofilm formation and is mediated primarily by surface proteins encoded by genes such as *als5* in *C. auris*, which facilitate amyloid fibril formation and influence cell adhesion and biofilm development (Gruber et al., 2025). The adhesion protein encoded by *scf1* plays a key role in mediating intercellular attachment, aggregation, and biofilm formation (Wang et al., 2024). The core transcriptional regulators encoded by *efg1* and *bcr1* are master regulators of *C. auris* biofilm formation and are closely associated with biofilm morphogenesis, cell morphology, and the transition between commensal and pathogenic states (Ganguly and Mitchell, 2011; Glazier, 2022). Biofilm formation also requires fungal cell wall integrity, mediated by *mkc1*, which is activated by surface contact and presumably contributes to the invasion of solid surfaces (Kumamoto, 2005). Proteins such as *erg2* and *erg11*, encoded by *erg2* and *erg11*, respectively, contribute to ergosterol synthesis and biofilm formation, and are associated with drug resistance (Verma et al., 2025). RT-qPCR analysis showed that treatment with the SLB–POS combination significantly downregulated the expression of these biofilm- and ergosterol-related genes. However, these transcriptional changes indicate altered mRNA expression but do not directly demonstrate functional impairment of adhesion, biofilm formation, or hyphal development. Under planktonic conditions, these expression patterns support the concept that the drug combination may interfere with multiple processes associated with biofilm development and pathogenicity. However, functional verification is required to confirm these effects.

In addition, oxidative stress and ROS accumulation are widely linked to antifungal responses and fungal cell injury, including in biofilm-associated infections (Cao et al., 2024). ROS generation and oxidative stress have been documented to correlate with cellular damage and reduced microbial survival in multiple antimicrobial studies (Dryden, 2018; Zhang et al., 2020). Specific antimicrobial agents have also been demonstrated to induce oxidative stress, disrupt cell wall integrity, and increase intracellular ROS levels (Ding et al., 2022; Huang et al., 2020; Hwang et al., 2012). In this study, increased ROS levels were observed concurrently with the enhanced antifungal effects of the SLB-POS combination under planktonic conditions. However, since functional verification using ROS scavengers was not performed, the observed ROS elevation should be considered a concomitant phenomenon rather than a definitive mediator of the synergistic antifungal activity. Whether similar ROS accumulation occurs in biofilm-associated cells and whether it contributes to the antibiofilm effect of the combination remain to be investigated.

The safety profile of the SLB–POS combination is another important consideration for clinical translation. Although SLB and POS each have well-established safety profiles, the cytotoxicity of the combination has not been evaluated directly in mammalian cells. SLB has been used clinically for decades as a hepatoprotective agent with a favorable safety profile and very low toxicity (Wu et al., 2024). The effective pharmacological concentration of SLB *in vivo* is approximately 0.2 μmol/L (0.0965 μg/mL) (Gillessen and Schmidt, 2020). In this study, the minimum concentration of SLB required to exert synergistic antifungal effects with POS was 4 μg/mL, which is higher than the plasma concentration typically achieved with conventional oral administration. Despite the extremely high safety margin of SLB, making dose escalation to reach this synergistic concentration theoretically relatively safe with limited acute toxicity risk, it cannot be ignored that the absolute oral bioavailability of SLB is low (approximately 0.45% ± 0.28%) due to its poor water solubility, limited membrane permeability, and active intestinal efflux (Li et al., 2025). Future studies should focus on improving its *in vivo* bioavailability and plasma concentration by optimizing administration routes and developing novel pharmaceutical formulations. In recent years, various novel drug delivery techniques have been developed and shown to significantly enhance the bioavailability of SLB, including nanotechnology-driven delivery systems, lipid carriers, water-soluble derivatives, bioavailability enhancers, intravenous formulations, and transdermal delivery systems, as well as controlled and controlled-release methods (Alkathiri et al., 2024; Li et al., 2025; Ma et al., 2017; Safarpour et al., 2022; Shangguan et al., 2015). These approaches have also been shown to improve tissue targeting, intracellular uptake, and therapeutic efficacy. Similarly, POS is an FDA-approved antifungal with well-documented safety data (Chen et al., 2020). Combination therapy may enable a reduction in the POS dose, potentially reducing azole-related side effects (Lecha et al., 2005), and the hepatoprotective properties of SLB may further counteract azole-associated liver toxicity. Therefore, before clinical translation, further *in vivo* verification, formulation optimization, and systematic toxicity evaluation (including acute and subacute toxicity tests) are needed to comprehensively assess the safety of this combination regimen. The *in vitro* findings of the synergistic antifungal activity of the SLB-POS combination in this study provide preliminary experimental evidence for subsequent *in vivo* pharmacodynamic studies and the development of clinical combination therapy strategies.

In conclusion, 10 clinical *C. auris* strains were tested in this study, with synergistic effects observed in seven isolates. This *in vitro* proof-of-concept study demonstrated that the combination of SLB and POS can inhibit biofilm formation, suppress biofilm development, and reduce the viability of pre-formed mature biofilms. The findings further suggest that the synergistic activity is associated with multiple biological events, including reduced ergosterol content, inhibition of biofilm formation, elevated oxidative stress, and altered expression of biofilm-related genes. These results provide preliminary experimental evidence supporting the further investigation of the SLB–POS combination as a potential therapeutic strategy against *C. auris* infections.

Despite these promising findings, this study has several limitations. All experiments were conducted *in vitro*, and verification of *in vivo* efficacy and safety using animal models was not performed. The observed changes in ergosterol content, gene expression, and ROS levels are only correlative, and functional studies are needed to confirm causal relationships. The mechanistic studies were limited to the seven synergistic strains identified in the checkerboard assay, potentially introducing selection bias and limiting the generalizability of the findings. It should also be noted that gene expression analyses were performed under planktonic conditions and may not fully reflect the gene expression profiles in biofilms. Furthermore, the minimum synergistic concentration of SLB exceeds typical oral plasma concentrations, potentially limiting clinical translation. Finally, although SLB and POS have established individual safety profiles, the cytotoxicity of their combination in mammalian cells has not been assessed. Future studies should include larger, more diverse strain collections; *in vivo* efficacy and safety evaluations; mechanistic validation; formulation optimization to improve SLB bioavailability; and comprehensive toxicity assessments to support clinical translation.

## Data Availability

The datasets presented in this study can be found in online repositories. The names of the repository/repositories and accession number(s) can be found in the article/Supplementary material.
