# Supplementary material for: In vitro synergistic antifungal activity of silybin-posaconazole combination against Candida auris
Source: Front Microbiol. 2026 Oct 21;17:1881511. doi: 10.3389/fmicb.2026.1881511 (PMC13731284; doi:10.3389/fmicb.2026.1881511)
Supplement: Supplementary file 1 [file Table_1.DOCX]

Table S1. Combined susceptibility test results of SLB with ITR, VOR, POS, and FLC

| **Strains** | **MICofdrug(μg/mL)** | | | | | **MIC[SLB/Combination(μg/mL)]（FICI）** | | | |
| --- | --- | --- | --- | --- | --- | --- | --- | --- | --- |
|  | **Alone** | | | | | **Combination** | | | |
|  | **SLB** | **ITR** | **VOR** | **POS** | **FLC** | **SLB/ITR/FICI** | **SLB/VOR/FICI** | **SLB/POS/FICI** | **SLB/FLC/FICI** |
| ATCC 22019 | >128 | 0.25 | 1 | 0.5 | 2 | 1/0.25/1.004(I) | 4/0.5/0.516(I) | 1/0.5/1.004(I) | 8/1/0.531(I) |
| 381 | >128 | 0.5 | 2 | 0.125 | 8 | 8/0.5/1.031(I) | 8/1/0.531(I) | <1/0.125/1.002(I) | 128/8/1.5(I) |
| 382 | >128 | 0.5 | 1 | 0.5 | >8 | 2/2/4.008(A) | <1/0.5/0.502(I) | <1/0.5/1.002(I) | 64/8/0.75(I) |
| 383 | >128 | 1 | 4 | 0.5 | >8 | 8/1/1.031(I) | 4/0.5/0.141(S) | 4/0.125/0.266(S) | 64/8/0.75 (I) |
| 384 | >128 | 2 | 1 | 0.5 | 8 | 8/0.25/0.156(S) | 1/0.125/0.129(S) | 16/0.25/0.563(I) | 64/8/1.25(I) |
| 385 | >128 | 1 | 8 | 1 | >8 | 8/0.25/0.281(S) | 4/4/0.516(I) | 2/0.0625/0.070(S) | 64/8/0.75(I) |
| 386 | >128 | 2 | >8 | 1 | >8 | 8/1/0.531(I) | 16/8/0.563(I） | 4/0.125/0.141(S) | 64/8/0.75(I) |
| 387 | >128 | 1 | 1 | 0.5 | >8 | 4/0.25/0.266(S) | 2/0.125/0.133(S) | 2/0.125/0.258(S) | 64/8/0.75(I) |
| 388 | >128 | 2 | 1 | 0.5 | >8 | 32/1/0.625(I) | 32/0.25/0.375(S) | 8/0.125/0.281(S) | 64/8/0.75 (I) |
| 389 | >128 | 1 | 4 | 0.25 | >8 | 8/0.5/0.531(I) | 32/0.5/0.25(S) | 8/0.0625/0.281(S) | 64/8/0.75(I) |
| 390 | >128 | 0.5 | 4 | 0.5 | >8 | 32/0.25/0.625(I） | 4/4/1.016 (I) | 8/0.125/0.281(S) | 64/8 /0.75(I) |

**Note:** ITR, itraconazole; VOR, voriconazole; POS, posaconazole; FLC, fluconazole； SLB, Silybin; S, synergism (FICI ≤0.5); I, indifference (no interaction, FICI from >0.5 to ≤4); A, antagonism (FICI >4). The MIC represented the concentration that resulted in 50% growth inhibition; FICI: fractional inhibitory concentration index; FICI = [MIC of Drug A (combination) / MIC of Drug A (alone)] + [MIC of Drug B (combination) / MIC of Drug B (alone)]; For FICI calculations, concentrations of 256, 16 and 0.5μg/mL were used when MICs were >128, >8 and <1 μg/mL, respectively.

Table S2. Effects of POS and SLB-POS on ergosterol content in eight C. auris strains

| **Strain** | **POS, mean ± SD (%)** | **POS - SLB, mean ± SD (%)** | **Reduction, POS / POS - SLB, pp (Šidák-adjusted 95% CI)** | **Šidák-adjusted *P*** | **Strain** | **POS, mean ± SD (%)** |
| --- | --- | --- | --- | --- | --- | --- |
| 382 | 9.807 ± 0.432 | 10.425 ± 0.279 | −0.618 (−1.254 to 0.018) | 0.0609 | 382 | 9.807 ± 0.432 |
| 383 | 16.566 ± 0.069 | 10.423 ± 0.067 | 6.144 (5.508 to 6.780) | <0.0001 | 383 | 16.566 ± 0.069 |
| 385 | 10.302 ± 0.249 | 9.620 ± 0.066 | 0.683 (0.047 to 1.319) | 0.0291 | 385 | 10.302 ± 0.249 |
| 386 | 12.397 ± 0.328 | 8.271 ± 0.114 | 4.127 (3.491 to 4.763) | <0.0001 | 386 | 12.397 ± 0.328 |
| 387 | 13.420 ± 0.308 | 10.423 ± 0.067 | 2.998 (2.362 to 3.634) | <0.0001 | 387 | 13.420 ± 0.308 |
| 388 | 10.302 ± 0.249 | 8.584 ± 0.134 | 1.719 (1.083 to 2.355) | 4.25 × 10⁻⁸ | 388 | 10.302 ± 0.249 |
| 389 | 10.423 ± 0.067 | 9.570 ± 0.021 | 0.853 (0.217 to 1.489) | 0.00357 | 389 | 10.423 ± 0.067 |
| 390 | 11.331 ± 0.673 | 9.344 ± 0.207 | 1.988 (1.352 to 2.624) | 1.63 × 10⁻⁹ | 390 | 11.331 ± 0.673 |

Note：Values are means ± SD from three independent biological replicates. Ergosterol content was analyzed using two-way ANOVA with strain and treatment as fixed factors, including the strain × treatment interaction. Because the interaction was significant, POS and POS - SLB were compared within each strain using Šidák’s multiple-comparison correction across the eight strain-specific comparisons. Reductions are expressed in percentage points (pp), and adjusted 95% confidence intervals are reported as effect sizes. Partial η² is reported for the omnibus ANOVA effects.

Table S3. Effect of POS - SLB on biofilm inhibition in eight *C. auris* strains

| **Strain** | **POS, mean ± SD (%)** | **POS - SLB, mean ± SD (%)** | **Difference, POS - SLB/ POS, pp (Šidák-adjusted 95% CI)** | **Šidák-adjusted *P*** |
| --- | --- | --- | --- | --- |
| 382 | 48.277 ± 3.960 | 41.887 ± 1.831 | −6.390 (−19.842 to 7.062) | 0.7859 |
| 383 | 45.627 ± 7.989 | 74.137 ± 0.617 | 28.510 (15.058 to 41.962) | 5.09 × 10⁻⁶ |
| 385 | 15.500 ± 10.528 | 62.073 ± 2.424 | 46.573 (33.121 to 60.026) | 1.41 × 10⁻¹⁰ |
| 386 | 61.463 ± 0.369 | 79.740 ± 2.868 | 18.277 (4.824 to 31.729) | 0.00309 |
| 387 | 22.590 ± 3.638 | 58.367 ± 0.699 | 35.777 (22.324 to 49.229) | 6.01 × 10⁻⁸ |
| 388 | 37.340 ± 13.906 | 51.250 ± 0.520 | 13.910 (0.458 to 27.362) | 0.039 |
| 389 | 21.950 ± 7.789 | 38.497 ± 3.822 | 16.547 (3.094 to 29.999) | 0.0087 |
| 390 | 13.693 ± 4.337 | 24.197 ± 0.449 | 10.503 (−2.949 to 23.956) | 0.2131 |

Note：Values are means ± SD from three independent biological replicates. Biofilm inhibition was analyzed using two-way ANOVA with strain and treatment as fixed factors, including the strain × treatment interaction. Because the interaction was significant, within-strain comparisons between POS and POS - SLB were performed using Šidák’s multiple-comparison correction across the eight strain-specific tests. Differences are expressed in percentage points (pp), and adjusted 95% confidence intervals are reported as effect sizes. Partial η² is reported for the omnibus effects.

Table S4.Effects of POS - SLB on ROS Fluorescence Intensity in Eight C. auris Strains

| **Strain** | **None, mean ± SD** | **POS, mean ± SD** | **POS - SLB, mean ± SD** | **Difference, POS - SLB / POS (Tukey-adjusted 95% CI)** | **Tukey-adjusted *P*** |
| --- | --- | --- | --- | --- | --- |
| 382 | 1.0000 ± 0.0046 | 1.0374 ± 0.0177 | 1.0883 ± 0.0125 | 0.0509 (−0.0734 to 0.1752) | 0.5865 |
| 383 | 0.9983 ± 0.0082 | 1.0825 ± 0.1641 | 1.2981 ± 0.1692 | 0.2155 (0.0912 to 0.3398) | 0.00034 |
| 385 | 1.0014 ± 0.0155 | 1.0305 ± 0.0500 | 1.2696 ± 0.0645 | 0.2391 (0.1148 to 0.3634) | <0.0001 |
| 386 | 1.0017 ± 0.0086 | 1.0414 ± 0.0885 | 1.4031 ± 0.0798 | 0.3617 (0.2374 to 0.4860) | <0.0001 |
| 387 | 0.9970 ± 0.0080 | 0.9914 ± 0.0263 | 1.1065 ± 0.0321 | 0.1151 (−0.0092 to 0.2393) | 0.0748 |
| 388 | 1.0012 ± 0.0128 | 0.9967 ± 0.0627 | 1.1387 ± 0.0338 | 0.1420 (0.0177 to 0.2663) | 0.0217 |
| 389 | 1.0053 ± 0.0078 | 1.0271 ± 0.0335 | 1.2365 ± 0.0830 | 0.2094 (0.0851 to 0.3337) | 0.000497 |
| 390 | 1.0041 ± 0.0231 | 0.9918 ± 0.0113 | 1.3154 ± 0.0453 | 0.3236 (0.1993 to 0.4478) | <0.0001 |

Note：Values are means ± SD from three independent biological replicates. Two-way ANOVA was performed with strain and treatment as fixed factors, including their interaction. When the interaction was significant, Tukey-adjusted multiple-comparison tests were performed within each strain to compare POS with POS - SLB. Differences are expressed in arbitrary fluorescence units, with Tukey-adjusted 95% confidence intervals reported as effect sizes. Partial η² is reported for the omnibus ANOVA effects.

Table S5. Effects of POS - SLB on expression of biofilm-related genes in eight C. auris strains

| **Gene** | **Strain** | **POS (mean ± SD)** | **POS-SLB (mean ± SD)** | **Fold change (POS-SLB/POS)** | **log2FC** | **Mean difference (95% CI)** | **F** | **P** | **eta squared** |
| --- | --- | --- | --- | --- | --- | --- | --- | --- | --- |
| *bcr1* | 382 | 0.0368 ± 0.0278 | 0.0617 ± 0.0046 | 1.6775 | 0.746 | -0.0249 (-0.2341 to 0.1842) | 0.059 | 0.8098 | 0.002 |
| *efg1* | 382 | 0.4504 ± 0.1364 | 0.3468 ± 0.0467 | 0.7699 | -0.377 | 0.1037 (-0.0268 to 0.2341) | 2.618 | 0.1155 | 0.076 |
| *scf1* | 382 | 0.0078 ± 0.0028 | 0.0212 ± 0.0108 | 2.7162 | 1.442 | -0.0134 (-0.1481 to 0.1213) | 0.041 | 0.8407 | 0.001 |
| *mkc1* | 382 | 0.4466 ± 0.0499 | 0.0063 ± 0.0011 | 0.0142 | -6.137 | 0.4403 (0.3024 to 0.5782) | 42.277 | <0.0001 | 0.569 |
| *erg2* | 382 | 0.0788 ± 0.0089 | 0.0172 ± 0.0038 | 0.218 | -2.197 | 0.0616 (-0.0811 to 0.2044) | 0.774 | 0.3855 | 0.024 |
| *erg11* | 382 | 0.0864 ± 0.0515 | 0.0066 ± 0.0016 | 0.0768 | -3.703 | 0.0797 (-0.0932 to 0.2526) | 0.882 | 0.3547 | 0.027 |
| *als5* | 382 | 0.3209 ± 0.0754 | 0.0331 ± 0.0086 | 0.1033 | -3.276 | 0.2878 (0.1291 to 0.4464) | 13.647 | 0.0008 | 0.299 |
| *bcr1* | 383 | 0.2375 ± 0.0088 | 0.0121 ± 0.0008 | 0.0509 | -4.295 | 0.2254 (0.0162 to 0.4345) | 4.818 | 0.0355 | 0.131 |
| *efg1* | 383 | 0.5145 ± 0.0178 | 0.0094 ± 0.0010 | 0.0183 | -5.772 | 0.5051 (0.3746 to 0.6356) | 62.172 | <0.0001 | 0.66 |
| *scf1* | 383 | 0.5217 ± 0.0945 | 0.0063 ± 0.0020 | 0.012 | -6.376 | 0.5154 (0.3807 to 0.6501) | 60.765 | <0.0001 | 0.655 |
| *mkc1* | 383 | 0.4445 ± 0.0071 | 0.0252 ± 0.0023 | 0.0566 | -4.143 | 0.4193 (0.2814 to 0.5572) | 38.343 | <0.0001 | 0.545 |
| *erg2* | 383 | 0.4807 ± 0.0051 | 0.0129 ± 0.0009 | 0.0267 | -5.225 | 0.4678 (0.3251 to 0.6105) | 44.59 | <0.0001 | 0.582 |
| *erg11* | 383 | 0.5600 ± 0.0204 | 0.2943 ± 0.0214 | 0.5256 | -0.928 | 0.2657 (0.0928 to 0.4386) | 9.798 | 0.0037 | 0.234 |
| *als5* | 383 | 0.5460 ± 0.0246 | 0.0537 ± 0.0032 | 0.0984 | -3.346 | 0.4923 (0.3336 to 0.6510) | 39.936 | <0.0001 | 0.555 |
| *bcr1* | 385 | 0.8119 ± 0.1665 | 0.1156 ± 0.0254 | 0.1424 | -2.812 | 0.6962 (0.4871 to 0.9054) | 45.981 | <0.0001 | 0.59 |
| *efg1* | 385 | 0.3284 ± 0.0272 | 0.1231 ± 0.0209 | 0.3749 | -1.415 | 0.2053 (0.0748 to 0.3358) | 10.27 | 0.0031 | 0.243 |
| *scf1* | 385 | 0.3652 ± 0.1583 | 0.0335 ± 0.0085 | 0.0917 | -3.446 | 0.3317 (0.1970 to 0.4663) | 25.164 | <0.0001 | 0.44 |
| *mkc1* | 385 | 0.3573 ± 0.0609 | 0.0238 ± 0.0036 | 0.0665 | -3.909 | 0.3335 (0.1956 to 0.4714) | 24.254 | <0.0001 | 0.431 |
| *erg2* | 385 | 0.6422 ± 0.1790 | 0.0643 ± 0.0297 | 0.1002 | -3.319 | 0.5778 (0.4351 to 0.7205) | 68.022 | <0.0001 | 0.68 |
| *erg11* | 385 | 0.2624 ± 0.0254 | 0.0255 ± 0.0087 | 0.0971 | -3.365 | 0.2369 (0.0640 to 0.4098) | 7.788 | 0.0088 | 0.196 |
| *als5* | 385 | 0.2553 ± 0.0395 | 0.0139 ± 0.0062 | 0.0544 | -4.199 | 0.2414 (0.0827 to 0.4001) | 9.602 | 0.004 | 0.231 |
| *bcr1* | 386 | 0.8937 ± 0.0669 | 0.3209 ± 0.0323 | 0.359 | -1.478 | 0.5728 (0.3637 to 0.7819) | 31.122 | <0.0001 | 0.493 |
| *efg1* | 386 | 0.8832 ± 0.2532 | 0.0074 ± 0.0011 | 0.0084 | -6.891 | 0.8758 (0.7453 to 1.0063) | 186.896 | <0.0001 | 0.854 |
| *scf1* | 386 | 0.3296 ± 0.0829 | 0.0010 ± 0.0001 | 0.0031 | -8.328 | 0.3285 (0.1939 to 0.4632) | 24.692 | <0.0001 | 0.436 |
| *mkc1* | 386 | 0.3549 ± 0.0610 | 0.0003 ± 0.0001 | 0.0007 | -10.4 | 0.3547 (0.2167 to 0.4926) | 27.433 | <0.0001 | 0.462 |
| *erg2* | 386 | 0.8301 ± 0.1816 | 0.0042 ± 0.0008 | 0.005 | -7.633 | 0.8259 (0.6832 to 0.9686) | 138.969 | <0.0001 | 0.813 |
| *erg11* | 386 | 0.7313 ± 0.1793 | 0.0015 ± 0.0005 | 0.0021 | -8.921 | 0.7298 (0.5569 to 0.9027) | 73.909 | <0.0001 | 0.698 |
| *als5* | 386 | 0.7667 ± 0.2001 | 0.0026 ± 0.0009 | 0.0034 | -8.219 | 0.7641 (0.6054 to 0.9228) | 96.214 | <0.0001 | 0.75 |
| *bcr1* | 387 | 0.5170 ± 0.0144 | 0.0013 ± 0.0004 | 0.0026 | -8.614 | 0.5157 (0.3065 to 0.7248) | 25.222 | <0.0001 | 0.441 |
| *efg1* | 387 | 0.8599 ± 0.0792 | 0.0030 ± 0.0007 | 0.0034 | -8.185 | 0.8570 (0.7265 to 0.9875) | 178.95 | <0.0001 | 0.848 |
| *scf1* | 387 | 0.6458 ± 0.0643 | 0.0020 ± 0.0007 | 0.0031 | -8.318 | 0.6438 (0.5091 to 0.7785) | 94.823 | <0.0001 | 0.748 |
| *mkc1* | 387 | 0.6034 ± 0.0410 | 0.0041 ± 0.0018 | 0.0068 | -7.2 | 0.5993 (0.4614 to 0.7373) | 78.34 | <0.0001 | 0.71 |
| *erg2* | 387 | 0.8478 ± 0.0396 | 0.0023 ± 0.0006 | 0.0027 | -8.535 | 0.8455 (0.7028 to 0.9882) | 145.648 | <0.0001 | 0.82 |
| *erg11* | 387 | 0.7523 ± 0.1033 | 0.0025 ± 0.0006 | 0.0033 | -8.224 | 0.7498 (0.5769 to 0.9227) | 78.015 | <0.0001 | 0.709 |
| *als5* | 387 | 0.6751 ± 0.0630 | 0.2910 ± 0.0844 | 0.431 | -1.214 | 0.3842 (0.2255 to 0.5428) | 24.32 | <0.0001 | 0.432 |
| *bcr1* | 388 | 0.8809 ± 0.1787 | 0.0019 ± 0.0006 | 0.0021 | -8.889 | 0.8791 (0.6699 to 1.0882) | 73.301 | <0.0001 | 0.696 |
| *efg1* | 388 | 0.6907 ± 0.0480 | 0.0049 ± 0.0015 | 0.007 | -7.15 | 0.6858 (0.5553 to 0.8163) | 114.604 | <0.0001 | 0.782 |
| *scf1* | 388 | 0.6457 ± 0.2390 | 0.1083 ± 0.0212 | 0.1676 | -2.576 | 0.5375 (0.4028 to 0.6721) | 66.082 | <0.0001 | 0.674 |
| *mkc1* | 388 | 0.8868 ± 0.2666 | 0.3196 ± 0.0843 | 0.3604 | -1.472 | 0.5672 (0.4293 to 0.7051) | 70.165 | <0.0001 | 0.687 |
| *erg2* | 388 | 0.5992 ± 0.0414 | 0.0153 ± 0.0036 | 0.0255 | -5.293 | 0.5840 (0.4413 to 0.7267) | 69.473 | <0.0001 | 0.685 |
| *erg11* | 388 | 0.8762 ± 0.2020 | 0.1253 ± 0.0382 | 0.143 | -2.806 | 0.7509 (0.5780 to 0.9238) | 78.26 | <0.0001 | 0.71 |
| *als5* | 388 | 0.5852 ± 0.2762 | 0.0093 ± 0.0020 | 0.0159 | -5.978 | 0.5759 (0.4173 to 0.7346) | 54.662 | <0.0001 | 0.631 |
| *bcr1* | 389 | 0.8641 ± 0.4253 | 0.0002 ± 0.0001 | 0.0002 | -12.38 | 0.8639 (0.6548 to 1.0730) | 70.793 | <0.0001 | 0.689 |
| *efg1* | 389 | 0.1561 ± 0.0117 | 0.0996 ± 0.0467 | 0.6382 | -0.648 | 0.0565 (-0.0740 to 0.1870) | 0.777 | 0.3846 | 0.024 |
| *scf1* | 389 | 0.2062 ± 0.0325 | 0.1609 ± 0.0213 | 0.7805 | -0.358 | 0.0453 (-0.0894 to 0.1799) | 0.468 | 0.4986 | 0.014 |
| *mkc1* | 389 | 0.7637 ± 0.1307 | 0.0031 ± 0.0011 | 0.0041 | -7.93 | 0.7605 (0.6226 to 0.8985) | 126.145 | <0.0001 | 0.798 |
| *erg2* | 389 | 0.9260 ± 0.2088 | 0.0087 ± 0.0062 | 0.0094 | -6.733 | 0.9173 (0.7746 to 1.0600) | 171.426 | <0.0001 | 0.843 |
| *erg11* | 389 | 0.7576 ± 0.2462 | 0.0037 ± 0.0008 | 0.0048 | -7.693 | 0.7539 (0.5810 to 0.9268) | 78.888 | <0.0001 | 0.711 |
| *als5* | 389 | 0.9935 ± 0.0138 | 0.0016 ± 0.0005 | 0.0016 | -9.255 | 0.9918 (0.8332 to 1.1505) | 162.113 | <0.0001 | 0.835 |
| *bcr1* | 390 | 0.4119 ± 0.0722 | 0.0045 ± 0.0007 | 0.0109 | -6.516 | 0.4074 (0.1983 to 0.6165) | 15.744 | 0.0004 | 0.33 |
| *efg1* | 390 | 0.3945 ± 0.0350 | 0.0043 ± 0.0009 | 0.0109 | -6.523 | 0.3902 (0.2597 to 0.5207) | 37.104 | <0.0001 | 0.537 |
| *scf1* | 390 | 0.1125 ± 0.0228 | 0.0751 ± 0.0109 | 0.6672 | -0.584 | 0.0375 (-0.0972 to 0.1721) | 0.321 | 0.575 | 0.01 |
| *mkc1* | 390 | 0.2963 ± 0.0556 | 0.0058 ± 0.0012 | 0.0194 | -5.684 | 0.2905 (0.1526 to 0.4284) | 18.405 | 0.0002 | 0.365 |
| *erg2* | 390 | 0.3039 ± 0.0698 | 0.0098 ± 0.0018 | 0.0324 | -4.948 | 0.2941 (0.1514 to 0.4368) | 17.62 | 0.0002 | 0.355 |
| *erg11* | 390 | 0.5734 ± 0.0852 | 0.6916 ± 0.1255 | 1.2062 | 0.271 | -0.1182 (-0.2912 to 0.0547) | 1.94 | 0.1732 | 0.057 |
| *als5* | 390 | 0.4541 ± 0.0948 | 0.1996 ± 0.0330 | 0.4397 | -1.186 | 0.2544 (0.0958 to 0.4131) | 10.668 | 0.0026 | 0.25 |

Note：Values represent relative gene expression. Each gene was analyzed separately using two-way ANOVA with strain and treatment as fixed factors, including the strain × treatment interaction. Only POS and POS - SLB were included in the inferential comparison; the None group was used solely for normalization. Within each strain, POS and POS - SLB were compared using Tukey’s multiple-comparison test. Fold changes were calculated as POS - SLB / POS; values <1 indicate down-regulation. The interaction was significant for all seven genes (F(7,32), P = 3.0 × 10⁻⁴ to 1.8 × 10⁻¹¹).

Table 6. Effects of POS and POS - SLB on XTT reduction in eight *C. auris* strains

| **Strain** | **POS, mean ± SD** | **POS - SLB, mean ± SD** | **Reduction, POS /POS - SLB (Šidák-adjusted 95% CI)** | **Šidák-adjusted *P*** |
| --- | --- | --- | --- | --- |
| 382 | 0.6724 ± 0.0427 | 0.6470 ± 0.0233 | 0.0254 (−0.0430 to 0.0939) | 0.9327 |
| 383 | 0.8959 ± 0.0055 | 0.7536 ± 0.0279 | 0.1423 (0.0738 to 0.2108) | 7.23 × 10⁻⁶ |
| 385 | 0.6853 ± 0.0263 | 0.4950 ± 0.0124 | 0.1903 (0.1218 to 0.2588) | 2.33 × 10⁻⁸ |
| 386 | 0.6874 ± 0.0203 | 0.5002 ± 0.0011 | 0.1872 (0.1187 to 0.2557) | 3.34 × 10⁻⁸ |
| 387 | 0.7422 ± 0.0148 | 0.4897 ± 0.0663 | 0.2525 (0.1840 to 0.3210) | 2.95 × 10⁻¹¹ |
| 388 | 0.5552 ± 0.0441 | 0.3347 ± 0.0361 | 0.2204 (0.1519 to 0.2889) | 8.21 × 10⁻¹⁰ |
| 389 | 0.4145 ± 0.0116 | 0.4337 ± 0.0142 | −0.0192 (−0.0877 to 0.0493) | 0.9871 |
| 390 | 0.4321 ± 0.0160 | 0.3640 ± 0.0180 | 0.0680 (−0.0005 to 0.1365) | 0.0524 |

Note：Values are means ± SD from three independent biological replicates. XTT reduction was analyzed using two-way ANOVA with strain and treatment as fixed factors, including the strain × treatment interaction. Because the interaction was significant, POS and POS - SLB were compared within each strain using Šidák’s multiple-comparison correction across the eight strain-specific comparisons. Differences are expressed in the original XTT reduction units, with Šidák-adjusted 95% confidence intervals reported as effect sizes. Partial η² is reported for the omnibus ANOVA effects.
